# ERS–PERK Pathway is Involved in the Repair of the Pulpo‐Dentinal Complex Under an Inflammatory Microenvironment

**DOI:** 10.1155/sci/7689998

**Published:** 2025-12-11

**Authors:** Yun Yang, Huimin Li, Yating Miao, Zhipu Luo, Lin Niu, Ruirui Liu

**Affiliations:** ^1^ Key Laboratory of Shaanxi Province for Craniofacial Precision Medicine Research, College of Stomatology, Xi’an Jiaotong University, Xi’an, Shaanxi, China, xjtu.edu.cn; ^2^ Clinical Research Center of Shaanxi Province for Dental and Maxillofacial Diseases, College of Stomatology, Xi’an Jiaotong University, Xi’an, Shaanxi, China, xjtu.edu.cn; ^3^ Department of Prosthodontics, College of Stomatology, Xi’an Jiaotong University, Xi’an, Shaanxi, China, xjtu.edu.cn; ^4^ Lanzhou University School of Stomatology, Lanzhou, Gansu, China

**Keywords:** ERS, injury repair, odontogenic differentiation, PERK, pulpo-dentinal complex

## Abstract

**Background:**

Protein kinase R‐like endoplasmic reticulum kinase (PERK) mediates the endoplasmic reticulum stress (ERS) response. However, it remains unclear whether PERK regulates the odontogenic differentiation of human dental pulp stem cells (hDPSCs) and contributes to the repair and regeneration of the pulpo‐dentinal complex (PDC) during inflammation. This study aimed to investigate the regulation of ERS–PERK in the differentiation and apoptosis of hDPSCs and its contribution to the repair and regeneration of PDC injury in the inflammatory microenvironment.

**Methods:**

In vivo dentin defect (DD group) and pulp perforation (PP group) were established to evaluate the expression and healing‐promoting properties of PERK in dental pulp at different injury stages. Using LPS with concentration gradients to simulate the inflammatory microenvironment, the activation of ERS–PERK pathway‐related genes was investigated using quantitative real‐time polymerase chain reaction (RT‐qPCR). hDPSCs’ apoptosis and odontogenic potential under inflammatory stimulation were also assessed using Calcein‐AM/7AAD Live/Dead cell double staining, RT‐qPCR, alkaline phosphatase (ALP) staining, and Alizarin Red S (ARS) staining. The potential role of PERK in odontogenesis and apoptosis of hDPSCs under inflammatory stimulation was explored.

**Results:**

With the extension of dental defects in vivo, PERK expression was gradually upregulated in the DD group, whereas, in the PP group, it increased in the early stage of inflammation and then decreased. Under LPS stimulation, the expression of inflammatory factors increased with the activation of the PERK pathway, while the ALP activity of hDPSCs and mineralized nodules decreased. PERK knockdown attenuated mRNA levels of ERS‐related genes and apoptosis‐related genes, whereas the expression of odontogenic‐related factors increased. The ALP activity and the number of mineralized nodules increased. PERK may regulate odontogenesis through the mitochondria‐associated endoplasmic reticulum (ER) membrane.

**Conclusion:**

Our results demonstrated PERK pathway activation, which enhances the expression of inflammatory factors and suppresses the odontogenic ability of hDPSCs in inflammatory microenvironments.

## 1. Introduction

The pulpo‐dentinal complex (PDC), a crucial biological unit of the tooth, is involved in immune defense and tissue repair against external stimuli [1]. However, persistent noxious stimuli, including bacteria, mechanical stimuli, and dental operations, have been shown to induce PDC injury and irreversible pulpitis, even leading to pulpal necrosis [2]. Although root canal treatment (RCT) is a classic therapy for inflamed and infected tooth pulp, there are still some postoperative complications, such as permanent devitalization and an increased risk of tooth fracture [1]. As cell biology and tissue engineering techniques in regenerative endodontics advance, functional regeneration of PDC by biological therapy has attracted significant interest in fundamental research and translational medicine.

In recent decades, dental pulp stem cells (DPSCs), with easy accessibility and odontogenic differentiation ability, have become promising seed cells for effectively maintaining PDC integrity and tooth vitality. The promotion of odontogenic differentiation is significant for PDC repair and regeneration. Persistent and long‐term detrimental stimuli are usually harmful to the intracellular homeostasis of DPSCs and associated with endoplasmic reticulum stress (ERS), including the inflammatory response, redox imbalance, and metabolic alterations. Hence, maintaining the intracellular homeostasis of DPSCs is critical under pathological conditions [3].

The double‐stranded RNA‐dependent protein kinase R‐like endoplasmic reticulum kinase (PERK) pathway, a major branch of the ERS pathway, functions as a double‐edged sword in cells. PERK dissociates from GRP78, a chaperone protein, phosphorylates eIF2α, and upregulates ATF4. Then, it reestablishes normal cellular function by attenuating protein translation and enhancing protein folding, antioxidant response, and amino acid biosynthesis [4, 5]. Unmitigated PERK signaling upregulates the proapoptotic transcription factor C/EBP‐homologous protein (CHOP), growth arrest, and DNA damage‐inducible 34 (GADD34). Excessive reactive oxygen species (ROS) and altered calcium homeostasis have been suggested to contribute to the opening of mitochondrial permeability transition pores (PTPs) and induce apoptosis [6].

PERK is involved in the osteodifferentiation of periodontal ligament cells (PDLCs), and its overexpression has been demonstrated to upregulate that of BSP and OCN, promoting osteogenesis of PDLCs [7]. In contrast, PERK knockdown inhibits osteodifferentiation and matrix mineralization [8]. However, it remains unclear whether the PERK pathway is involved in the injury response of pulp‐derived cells (PDCs). The exact contribution of the ERS–PERK pathway to the odontoblastic differentiation of human DPSCs (hDPSCs) under inflammatory conditions and the specific mechanisms involved remain to be fully elucidated.

This study aimed to investigate the function of the ERS–PERK pathway in the differentiation and biological behavior of hDPSCs at different stages of the inflammatory microenvironment, elucidate the regulatory mechanisms of ERS–PERK involved in the repair and regeneration of PDC, and propose novel strategies for the treatment of pulpitis.

## 2. Materials and Methods

### 2.1. Construction of Rat PDC Injury Models

All animal experiments were approved by the Ethics Committee of Xi’an Jiaotong University. Twenty‐four male Sprague–Dawley rats (4‐week‐old) weighing 180–220 g were anesthetized with 2% isoflurane inhalation. After sterilizing the maxillary molars with 75% ethanol, a 1 mm‐deep round cavity was prepared with a #1/4 round bur (TC‐21EF, Japan) on the occlusal surfaces of the left mandibular first molars as the dentin defect (DD) group; caution was exercised to avoid pulp exposure. In the pulp perforation group (PP group), a hole was drilled (conducted under water‐cooled conditions) in the central fossa of the occlusal surface of the maxillary first molars bilaterally. Most of the dentin was removed until only a thin layer remained showing a pinkish hue, at which point the drilling was stopped. A sterile explorer was used to probe and confirm pulp exposure points [9]. The pulp was penetrated by applying a slight pressure at the exposure point, resulting in mild bleeding. The area was rinsed with sterile saline and compressed with a sterile cotton pellet to achieve hemostasis. All procedures were performed under strict aseptic conditions by the same clinically experienced dentist. Surgeries were performed gently and efficiently to ensure airway patency and prevent suffocation. Postoperative monitoring included careful observation of the activity, sleep, and feeding behavior postrecovery; the rats were anesthetized with 2% isoflurane and performed heart perfusion with 4% paraformaldehyde (PFA) (Boster, China) buffered at pH 7.2–7.4 at 0, 1, 7, 14, and 21 days postsurgery.

### 2.2. Hematoxylin and Eosin (HE) Staining

The mandibles were removed and fixed in 4% PFA. Following decalcification with 10% ethylenediaminetetraacetic acid (EDTA) (pH = 7.4) (Boster, China) for 1 month, the tissues were embedded in paraffin and trimmed into 5‐μm‐thick sections by microtome (Leica, Germany). The sections were then dewaxed and stained using a HE staining kit (Solarbio, China) according to the manufacturer’s instructions.

### 2.3. Immunohistochemistry (IHC) Staining

IHC staining was performed using a Rabbit IgG IHC kit (Boster, China), according to the manufacturer’s instructions. PERK expression was detected with rabbit anti‐PERK as the primary antibody (1:200; Abcam) and completed with incubation with 3,3^′^‐diaminobenzidine (DAB) (ZSGB‐BIO, China).

### 2.4. hDPSCs Culture and Characterization

Healthy human dental pulp tissue was gently isolated from impacted third mandibular molars or premolars for orthodontic therapy from healthy donors (≤25 years old). Informed consent was obtained prior to sample collection. The study protocol was approved by the ethics committee of Stomatology Hospital of Xi’an Jiaotong University (Approval Number: [2022]005; date: May 10, 2022). Following isolation, pulp tissues were washed with PBS buffer (Gibco, USA) three times, cut into pieces, and digested with collagenase type I (Thermo, USA) for 40–60 min at 37°C. The cells were then seeded into a culture flask and cultured with alpha minimum essential medium (HyClone, USA) containing 10% fetal bovine serum (Gibco, USA) and 100 μg/mL penicillin/streptomycin (Gibco, USA) at 37°C under 5% CO_2_ humidified atmosphere. The medium was changed every 3 days, and hDPSCs at passages 2–5 were used for subsequent experiments. Flow cytometry was performed to identify hDPSCs phenotypes by screening the surface markers using the following antibodies: anti‐CD29 (SAB, USA), anti‐CD45 (SAB, USA), and anti‐CD90 (SAB, USA).

### 2.5. Calcein‐AM/7AAD Staining

hDPSCs were seeded in six‐well plates at a density of 2 × 10^5^ cells/well and treated with 0, 1, 10, 20, 30, 40, and 50 μg/mL LPS (Sigma, USA). After 24, 48, and 72 h, hDPSCs were harvested using 0.25% trypsin (Gibco), rinsed twice with PBS buffer, and then stained with 200 μL Calcein‐AM staining solution (Bestbio, China) and 200 μL 7‐AAD staining solution (Bestbio, China) at 37°C in the dark for 5–10 min, respectively. The apoptosis rate of hDPSCs was analyzed using flow cytometry (CytoFLFX SRT, China).

### 2.6. Real‐Time Polymerase Chain Reaction (PCR)

Total RNA from hDPSCs was extracted and reverse transcribed into cDNA using 5×Evo M‐MLV RT Reaction Mix (Accurate Biology, USA) following the manufacturer’s protocol. quantitative real‐time polymerase chain reaction (RT‐qPCR) was performed using a SYBR Green PCR kit (Accurate Biology, USA) according to the standard protocol. Denaturing, annealing, and extension conditions of each polymerase chain reaction cycle were 95°C for 30 s, 95°C for 5 s, and 60°C for 30 s, respectively. Primer sequences used for real‐time PCR are listed in Table 1.

**Table 1:** Sequences of the primers used for real‐time polymerase chain reaction.

Gene	Sequence (5′–3′)
*H-GAPDH-F*	TGTGTCCGTCGTGGATCTGA
*H-GAPDH-R*	TTGCTGTTGAAGTCGCAGGAG
*H-IL-1-F*	CCAGGGACAGGATATGGAGCA
*H-IL-1-R*	TTCAACACGCAGGACAGGTACAG
*H-TNF-α-F*	CTGCCTGCTGCACTTTGGAG
*H-TNF-α-R*	ACATGGGCTACAGGCTTGTCACT
*H-GRP78-F*	CTGTGCAGCAGGACATCAAGTTC
*H-GRP78-R*	TGTTTGCCCACCTCCAATATCA
*H-PERK-F*	AAGCACCACCAGAGAAGTGG
*H-PERK-R*	GTGCATCCATTGGGCTAGGA
*H-ATF4-F*	GGCCAAGCACTTCAAACCTC
*H-ATF4-R*	GACTGACCAACCCATCCACA
*H-chop-F*	CCGAGCTCTGATTGACCGAA
*H-chop-R*	AGCCACTTCTGGGAAAGGTG
*H-JNK-F*	TGAGCAGAAGCAAGCGTGAC
*H-JNK-R*	AGTCTGATTCTGAAATGGTCGG
*H-caspase-12-F*	TTGACCTTTTGGGGATGCGA
*H-caspase-12-R*	GCTTGGTCCCACAGATTCCA

### 2.7. Western Blot Analysis

hDPSCs were lysed with RIPA buffer (Beyotime, China) supplemented with PMSF (Beyotime, China) and protease inhibitor (Beyotime, China), and protein concentration was measured using the BCA protein assay kit (Beyotime, China). Protein samples were harvested, boiled, separated using polyacrylamide gel electrophoresis, and transferred onto polyvinylidene fluoride (PVDF) membranes (Millipore, Billerica USA, MA). The PVDF membranes were blocked with 5% milk and incubated with the following primary antibodies overnight at 4°C: anti‐PERK (Abcam; 1:500), anti‐DSPP (Santa Cruz Biotechnology; 1:500), anti‐RUNX2 (Santa Cruz Biotechnology; 1:1000), and anti‐GAPDH (weiao; 1:2000). On the following date, the membranes were washed with TBST and incubated with secondary antibodies (Boster, China) for 1 h at room temperature. Images were acquired using an ECL Hypersensitivity Luminescence Kit (Boster, China) and exposed to film.

### 2.8. Alkaline Phosphatase (ALP) and Alizarin Red S (ARS) Staining

hDPSCs were seeded in six‐well plates at a density of 2 × 10^5^ cells per well, cultured in *α*‐MEM medium containing 10% fetal bovine serum. After the cultures reached nearly 80% confluence, the medium was replaced with odontogenic medium (OM) containing 10% FBS (Gibco, USA), 1% penicillin/streptomycin (Gibco, USA), 10 mM sodium *β*‐glycerophosphate (Sigma, USA), 0.1 μM dexamethasone (Sigma, USA), and 50 μg/mL ascorbic acid (Sigma, USA) and supplemented with 1, 10, and 20 μg/mL LPS (Sigma, USA). The medium was changed every 2–3 days.

After 7 days, hDPSCs were stained using the BCIP/NBT ALP Colorimetric Kit according to the manufacturer’s instructions (Beyotime, China). ALP activity was measured using an ALP Activity Assay Kit (Beyotime, China) at an absorbance of 520 nm.

After 21 days, the hDPSCs were stained with ARS solution (Cyagen, USA). The stain was desorbed using 10% cetylpyridinium chloride (Solarbio, China), and the absorbance was quantified at 562 nm.

### 2.9. Cell Transfection of Lentivirus

The lentivirus for PERK knockdown was purchased from GenePharma Co. and added to the culture medium, following the manufacturer’s protocol. An empty GFP vector served as a negative control. Specifically, after hDPSCs were cultured to reach 40%–60% confluence, appropriate amounts of lentivirus according to the optimal multiplicity of infection (MOI) were transfected with polybrene. PERK expression was assessed using RT‐qPCR and western blotting.

### 2.10. RNA‐sequencing (RNA‐Seq) Analysis

Following PERK‐encoding lentivirus transfection, the hDPSCs were treated with 20 μg/mL LPS for 6 h. Total RNA was extracted using RNAiso Plus (Takara, Japan), used to generate cDNA libraries, and sequenced using an Illumina Novaseq 6000 (Shu Pu Biotechnologies LLC, Shanghai, China). Genes upregulated or downregulated more than two‐fold between the two groups and *p* value <0.05 were considered differentially expressed genes. The identified genes were used for Gene Ontology (GO) and Kyoto Encyclopedia of Genes and Genomes (KEGG) pathway analyses.

### 2.11. Statistical Analysis

All data were presented as mean ± standard deviation and analyzed using GraphPad Prism 8.0 for Windows. Student’s *t* test and one‐way analysis of variance (ANOVA) were used to analyze intra and intergroup differences, respectively. *p* < 0.05 was considered statistically significant.

## 3. Results

### 3.1. PERK Was Upregulated in Inflamed Rat Pulp Tissues

On the day of establishment of the DD group (D0), HE staining showed slightly congested capillaries, and thickened predentin appeared on D7 (Figure 1A). From D14 to D21, restorative dentin gradually formed beneath the cavity and narrowed the pulp cavity (Figure 1A). The inflammation progressed faster in the pulp exposure group. Telangiectasia and massive infiltration of inflammatory cells were observed on P1 (Figure 1A). On D14 and D21, the samples showed severe inflammation, resulting in inflammatory granulation tissue (Figure 1A).

Figure 1Upregulation of PERK in inflamed rat pulp tissues. (A) HE staining of the dental pulp (black arrow indicates a destructive lesion or restorative response). (B–D) IHC staining of PERK expression and quantitative analysis in rat dental pulp tissue at 0, 1, 7, 14, and 21 days post‐operation (the black arrow indicates positive staining of PERK).

**Figure:**
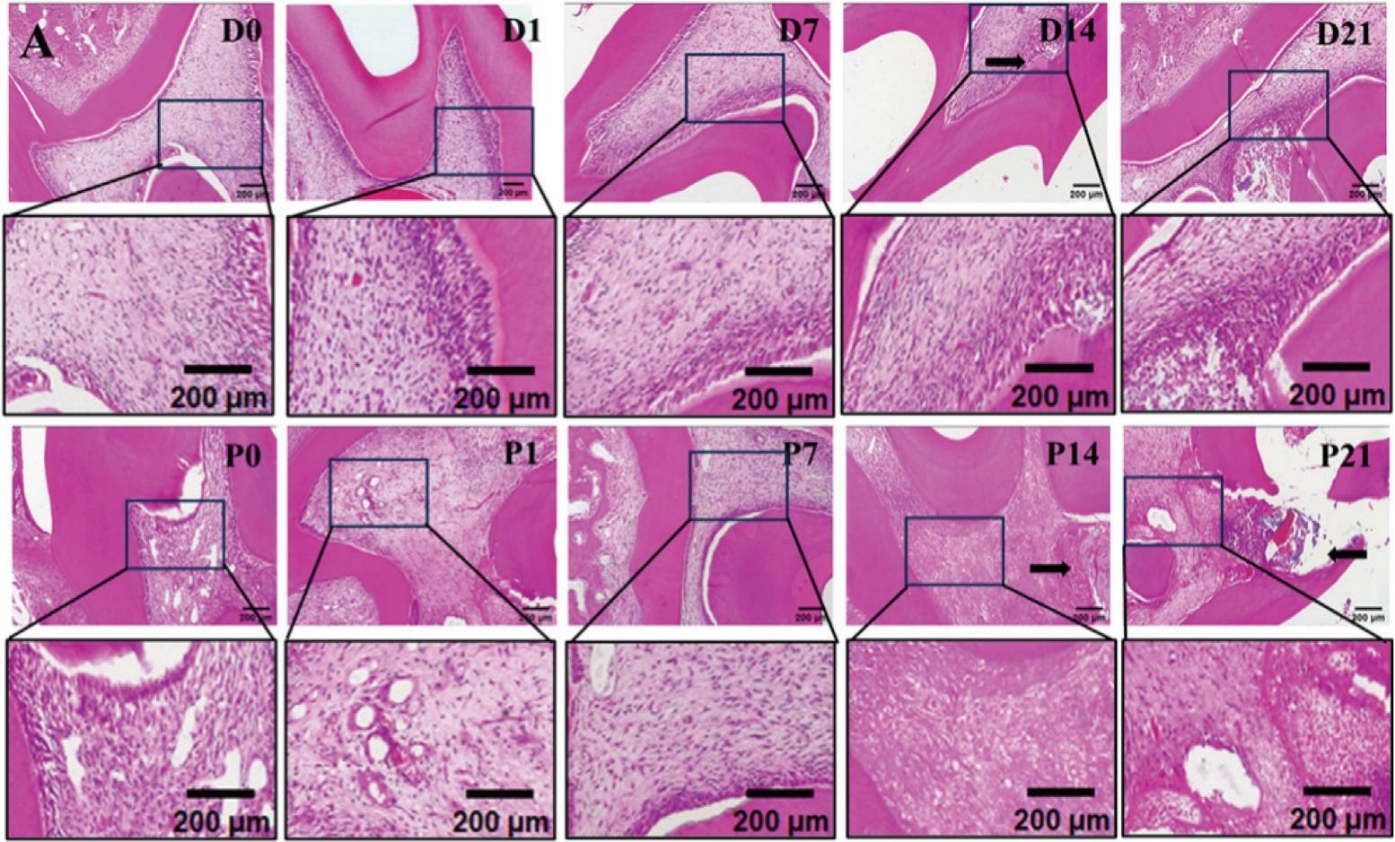
(A)

**Figure:**
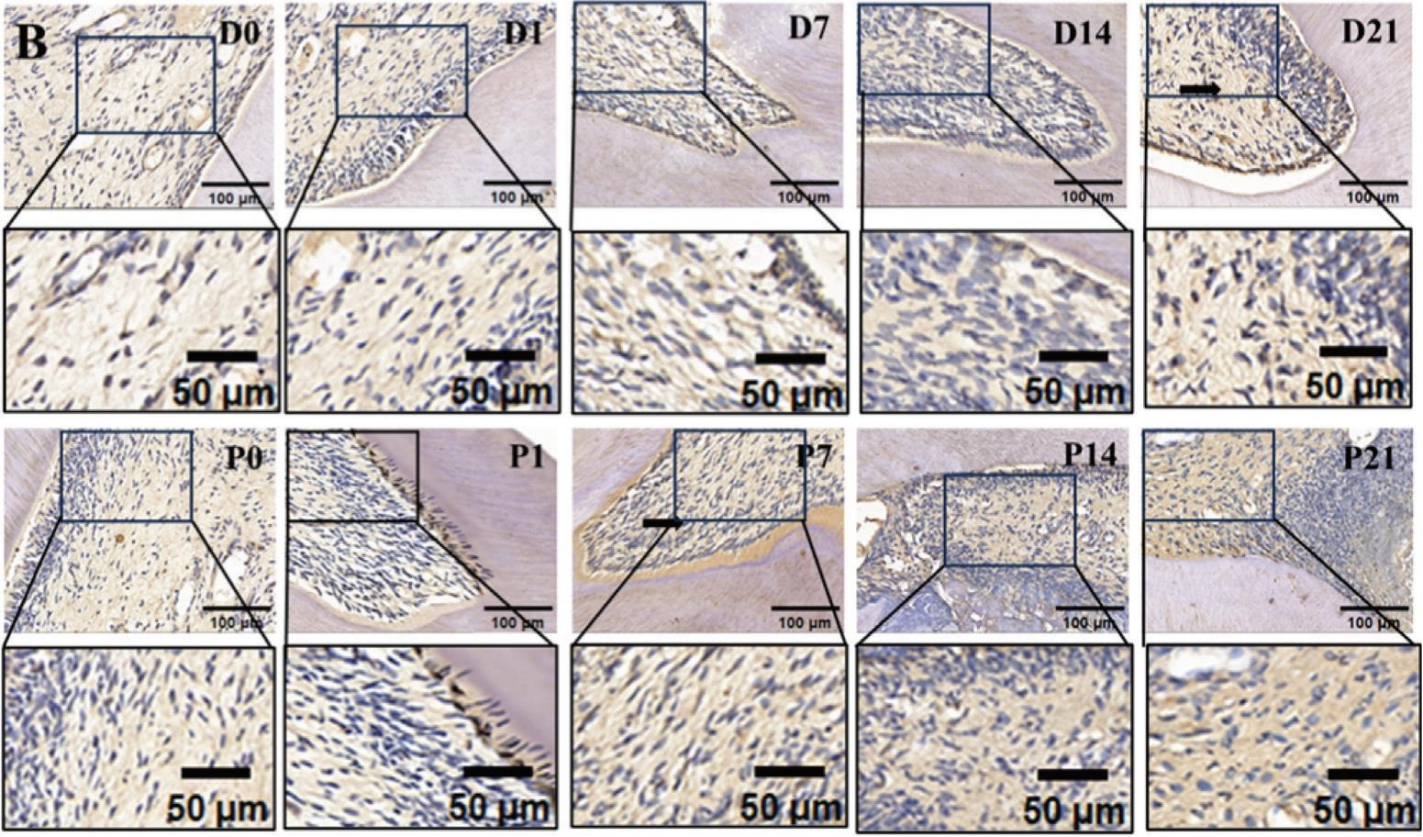
(B)

**Figure:**
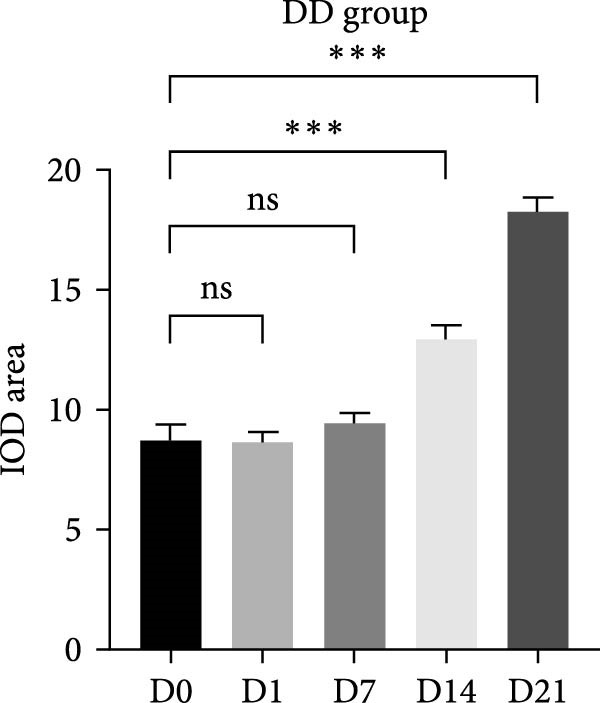
(C)

**Figure:**
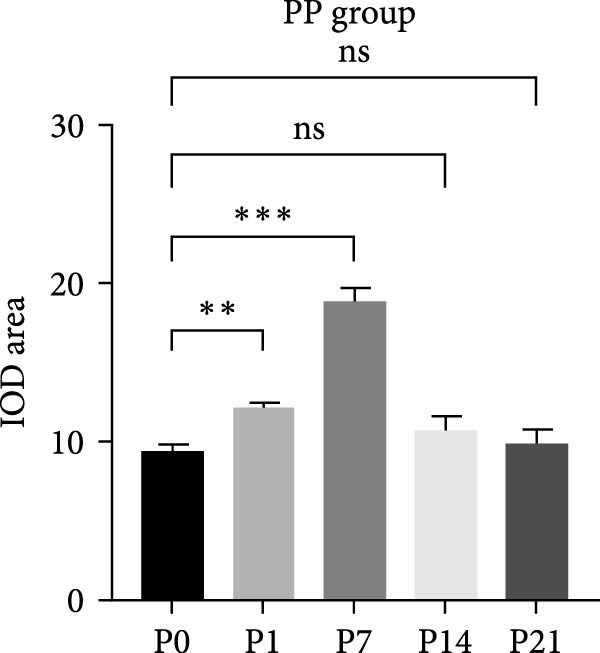
(D)

Immunohistochemical staining showed that PERK primarily appeared below the DD area at the early stages (D1 and D7) and remarkably increased with the extension of dentin exposure time (D14 and D21) (Figure 1B,C). In the pulp exposure group, although the positive staining of PERK in the pulp tissue significantly increased at the early stages (P1 and P7), it showed a downward trend after 14 days with pulp necrosis (Figure 1B,D).

### 3.2. The PERK Pathway Was Activated Under LPS‐Induced Inflammation

After primary culture for 7–10 days and passaging, the hDPSCs displayed a spindle or polygonal morphology, uniform size, consistent dimensions, and compact arrangement (Figure S1A,B). hDPSCs were identified using flow cytometry; the results indicated that CD29 and CD90 were highly expressed, whereas CD45 was barely detectable (Figure S1C). Stimulating hDPSCs with gradient concentration LPS (0, 1, 10, 20, 30, 40, and 50 μg/mL) for 24, 48, and 72 h showed that the survival rate was higher, peaking at 10 μg/mL at 24 h, and then showed a downward trend as the concentration of LPS and time increased (Figure 2A–C). The apoptosis rate was highest at a concentration of 40 μg/mL for 72 h (Figure 2C).

Figure 2ERS–PERK pathway activated under LPS‐induced inflammation. (A–C) Flow cytometry analysis of apoptosis of hDPSCs stimulated by different gradient concentration LPS (0, 1, 10, 20, 30, 40, and 50 μg/mL) for 24, 48, and 72 h. (D–G) The gene expression level of inflammatory cytokines (TNF‐*α* and IL‐1) and ERS‐related cytokine (PERK and ATF4) stimulated by different concentrations of LPS (0, 1, 10, or 20 μg/mL) for 6 h.

**Figure:**
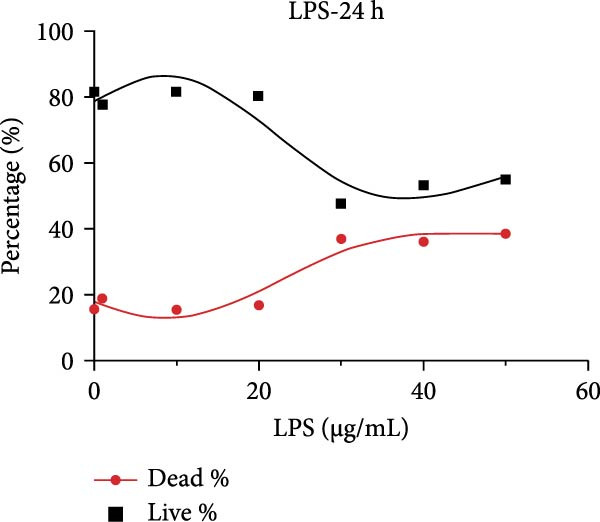
(A)

**Figure:**
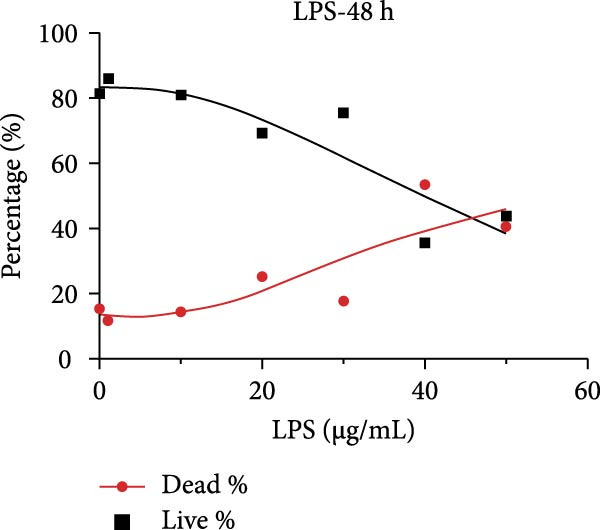
(B)

**Figure:**
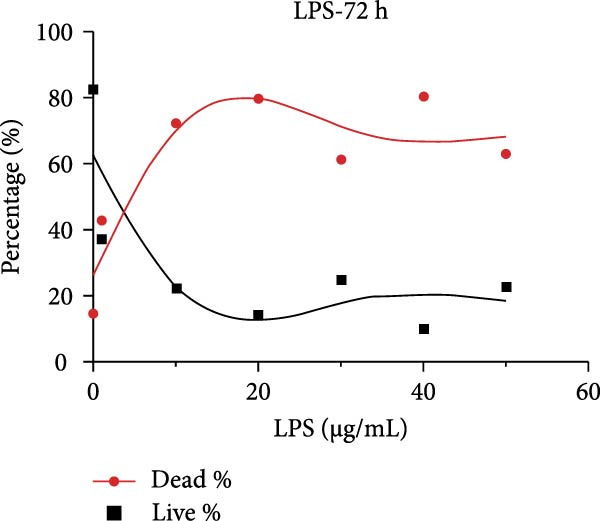
(C)

**Figure:**
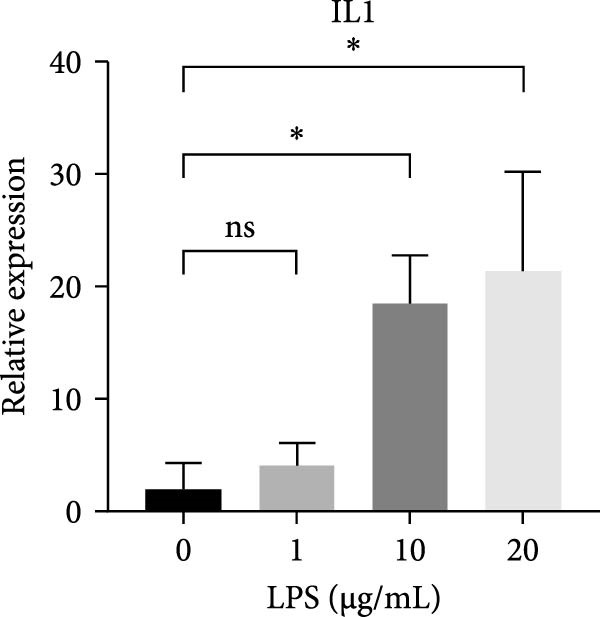
(D)

**Figure:**
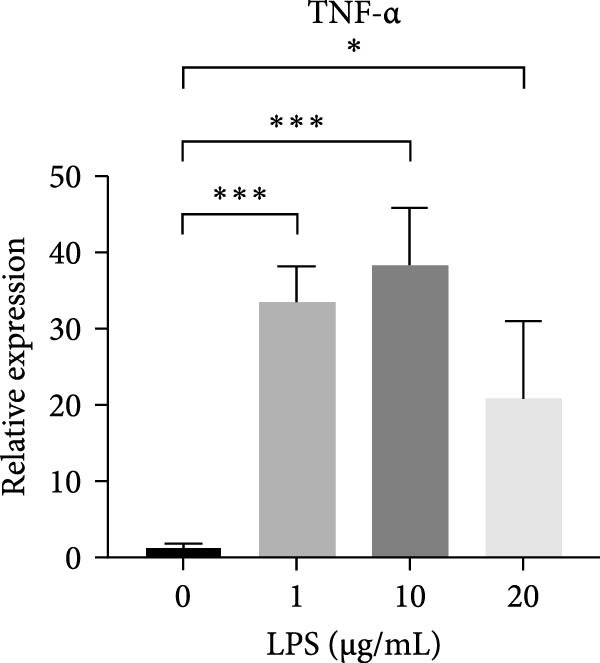
(E)

**Figure:**
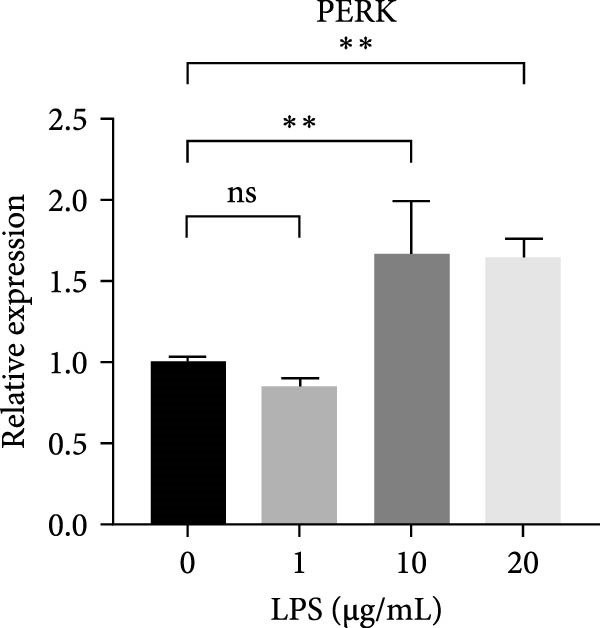
(F)

**Figure:**
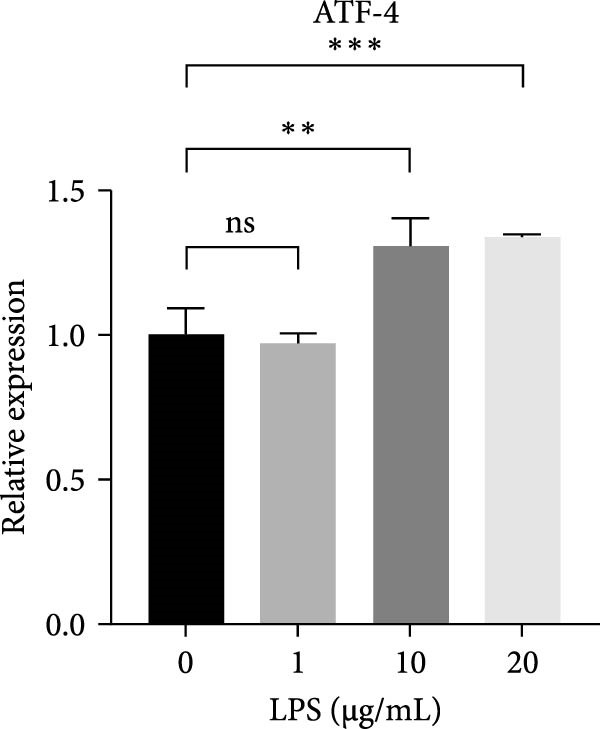
(G)

RT‐qPCR results showed that 1, 10, and 20 μg/mL LPS stimulation for 6 h increased the mRNA level of interleukin (IL)‐1 and tumor necrosis factor (TNF)‐*α* (Figure 2D,E). PERK showed a down trend at 1 μg/mL and LPS then increased at 10 and 20 μg/mL (Figure 2F) (*p* < 0.05), especially significantly increased in the 10 µg/mL group compared with other groups at 6 h (*p* < 0.05) (Figure 2F). ATF4, a downstream factor of PERK, also increased under 10 and 20 μg/mL LPS stimulation (Figure 2G) (*p* < 0.05). Further, hDPSCs were cultured in OM, which revealed that ALP activity was decreased under 10 and 20 μg/mL LPS stimulation compared with the OM group (*p* < 0.05) (Figure S2A B). ARS staining showed fewer mineralized nodules following LPS stimulation (*p* < 0.05) (Figure S2C,D). Therefore, 10 µg/mL LPS for 6 h was selected to establish the inflammation model for subsequent experiments.

### 3.3. PERK Suppressed hDPSC Odontogenic Differentiation Under LPS‐Induced Inflammation

hDPSCs were transfected by lentivirus at MOI of 1, 10, 20, 50, and 100 with or without 10 μg/mL polybrene. Green fluorescence was detected at 72 h posttransfection (Figure S3). At MOI = 50 with polybrene, the transfection efficiency of cells exceeded 80%; therefore, this condition was selected for subsequent experiments (Figure S3).

PERK was significantly inhibited in the shPERK group (Figure 3A–C). PERK knockdown markedly decreased the expression of ERS‐related cytokine genes (ATF4), ERS chaperonin (GRP‐78), and apoptotic cytokines (CHOP, JNK, and caspase‐12) (Figure 3D–H). RUNX2, OCN, and DSPP mRNA and protein expression levels were upregulated under inflammatory conditions (*p* < 0.05) (Figure 3I–N). ALP activity and the formation of mineralized nodules were increased compared to those in the shPERK‐NC group (Figure 3O–R).

Figure 3PERK signaling pathway suppressed hDPSC osteogenesis under LPS‐induced inflammation. (A–C) Knockdown efficiency of PERK in hDPSCs detected using RT‐qPCR and western Blot. (D–H) Changes in the expression levels of ERS chaperonin (GRP‐78) and apoptosis cytokines (CHOP, JNK, and caspase‐12) after PERK knockdown measured using RT‐qPCR. (I–K) Expression levels of odontoblastic differentiation‐related genes DSPP, OCN, and RUNX2 measured using RT‐qPCR. (L–N) Expression of DSPP and RUNX2 proteins examined using western blotting. (O, P) ALP staining and quantitative analysis after culturing hDPSCs with PERK siRNA in OM for 7 days under inflammation. (Q, R) Alizarin red S staining and quantitative analysis after culturing hDPSCs in OM for 21 days with PERK siRNA under inflammation. Significance was determined via Student’s *t*‐test analysis of variance (*n* = 3); data are represented as mean ± SD.  ^∗^ *p* < 0.05.  ^∗∗^ *p* < 0.01.  ^∗∗∗^ *p* < 0.001.

**Figure:**
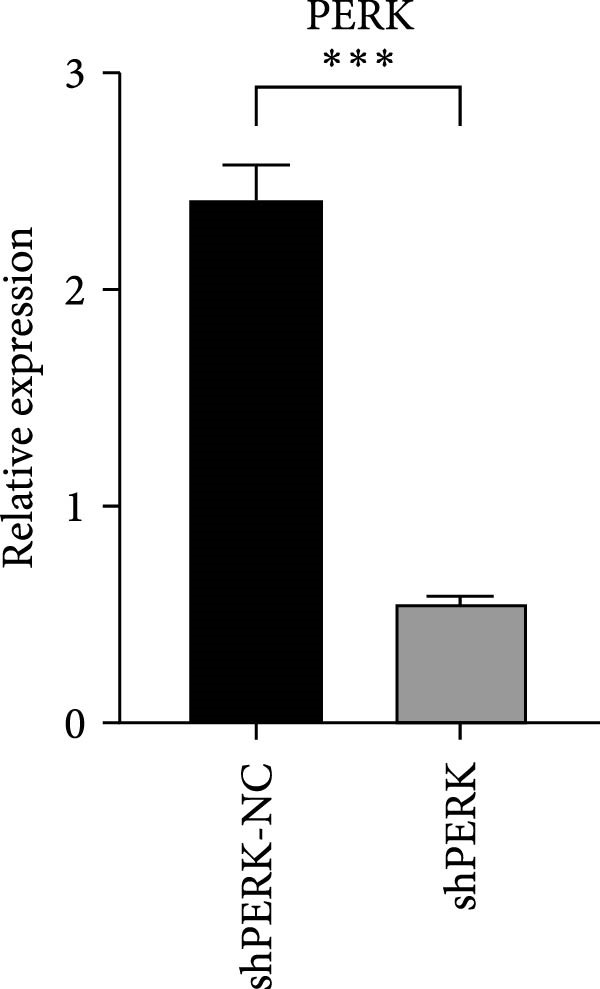
(A)

**Figure:**
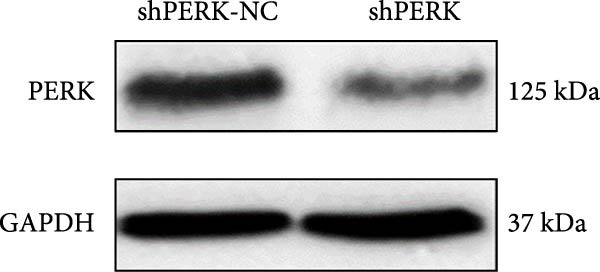
(B)

**Figure:**
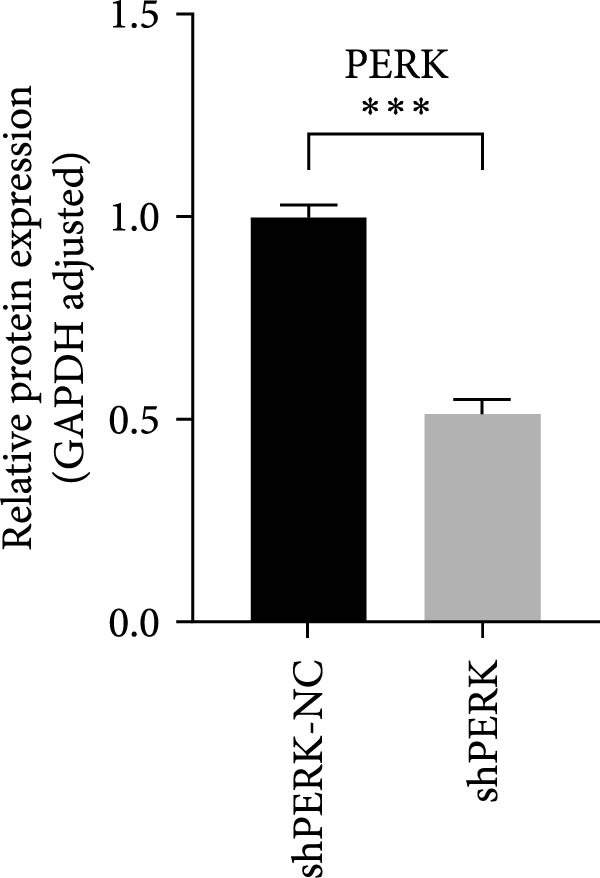
(C)

**Figure:**
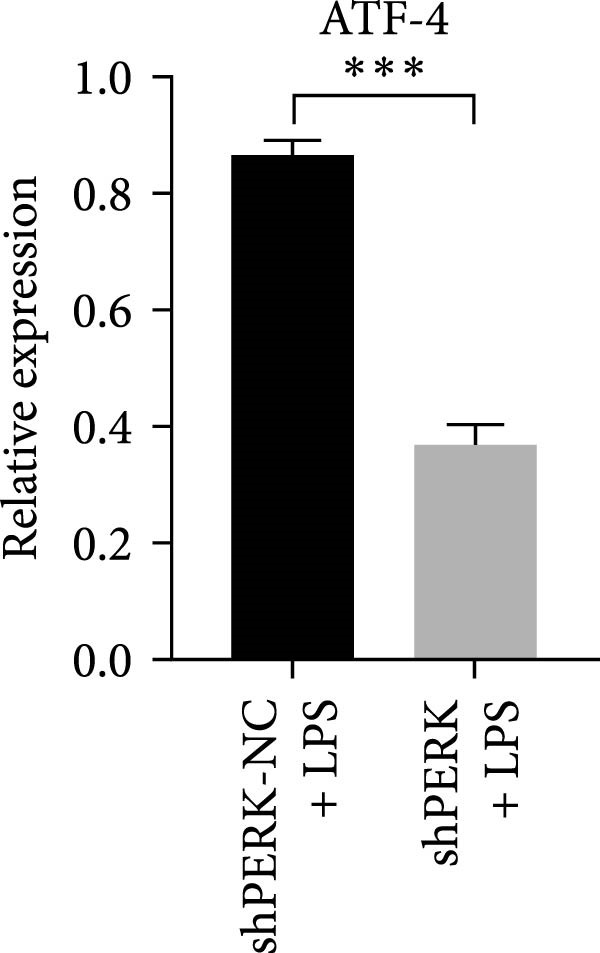
(D)

**Figure:**
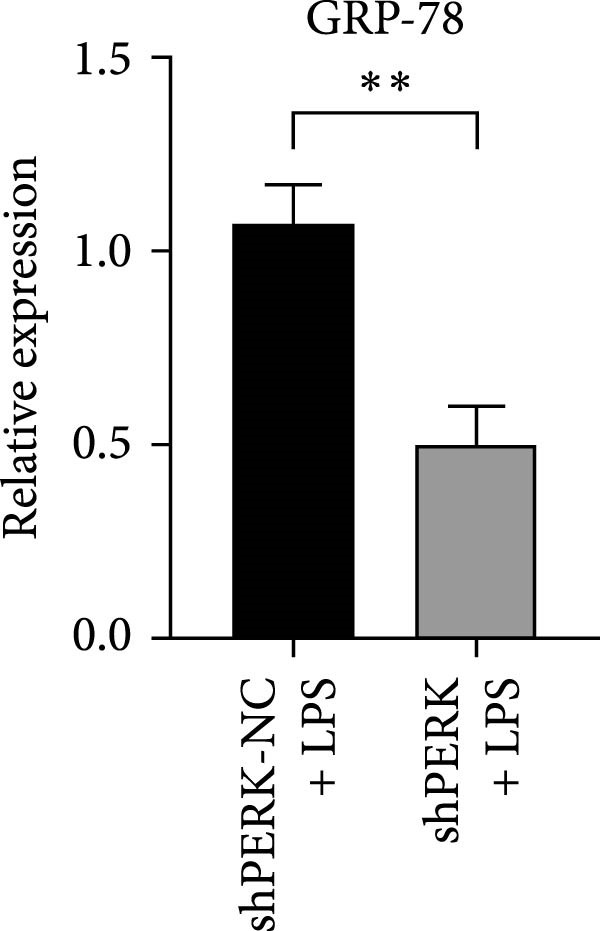
(E)

**Figure:**
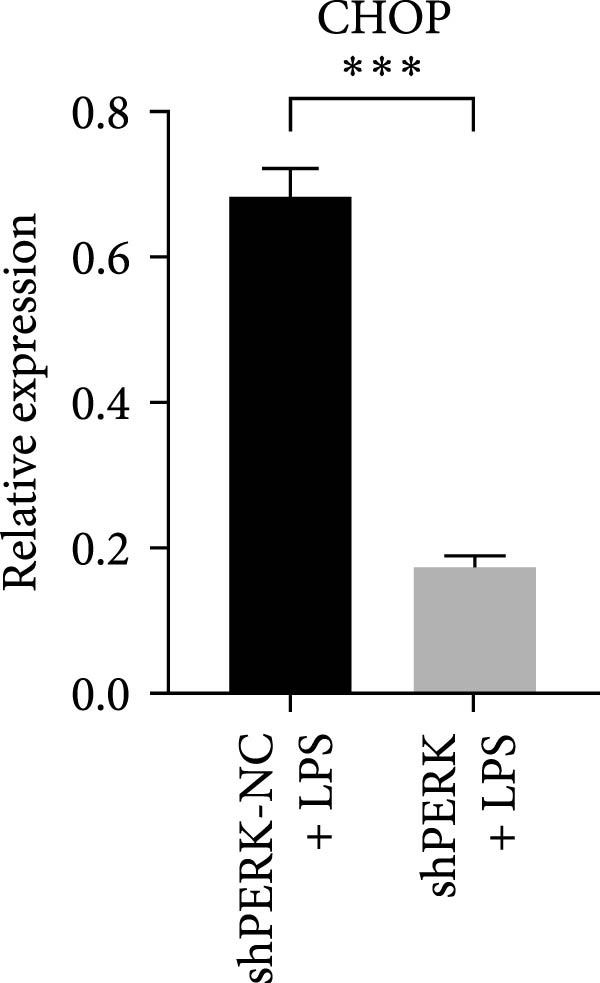
(F)

**Figure:**
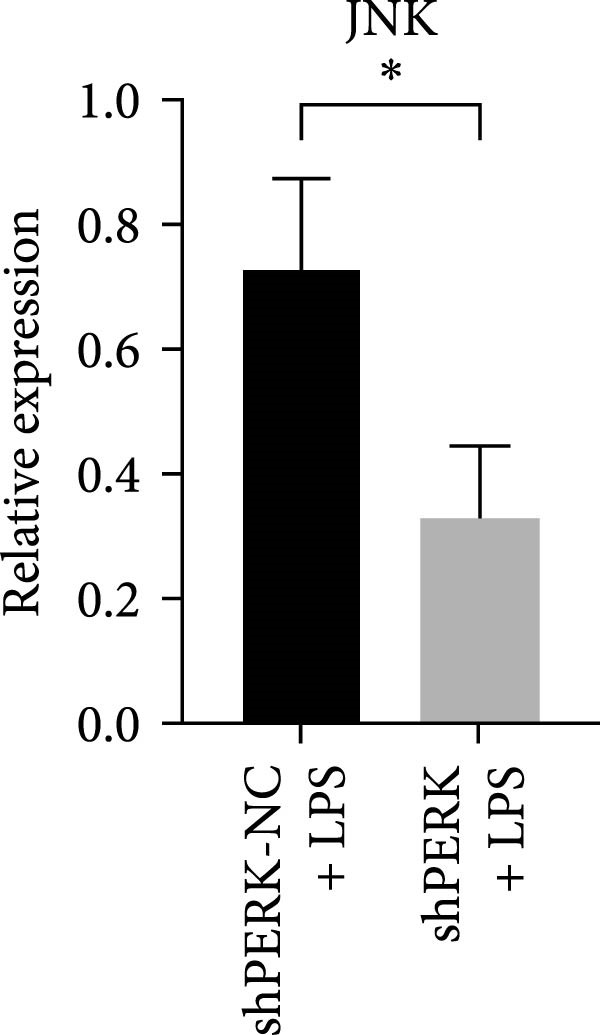
(G)

**Figure:**
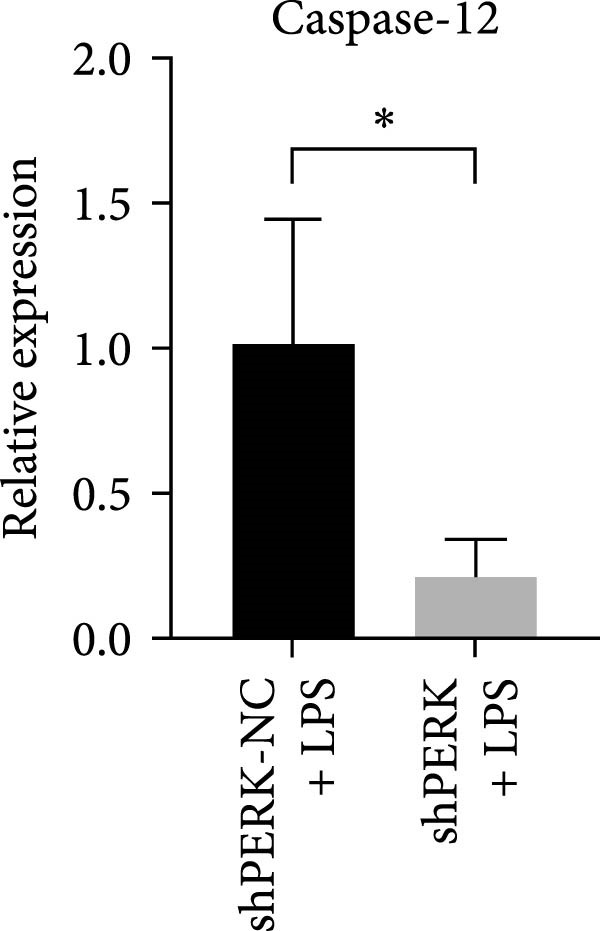
(H)

**Figure:**
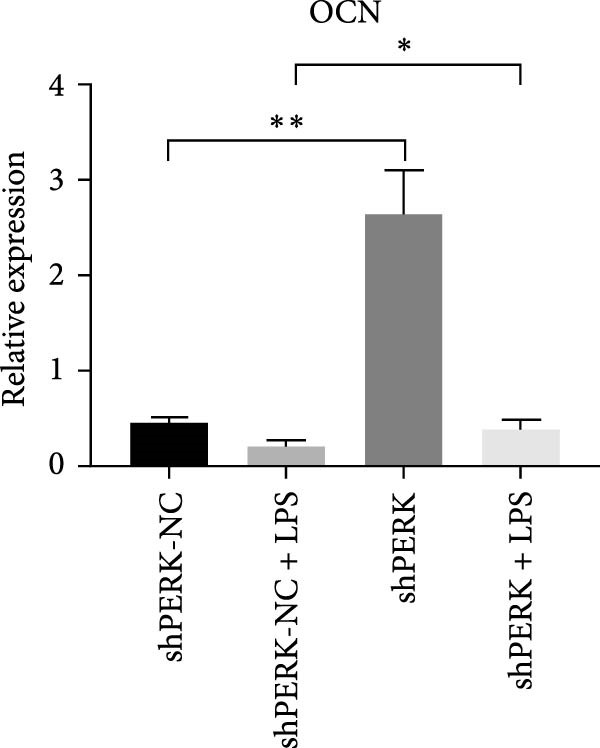
(I)

**Figure:**
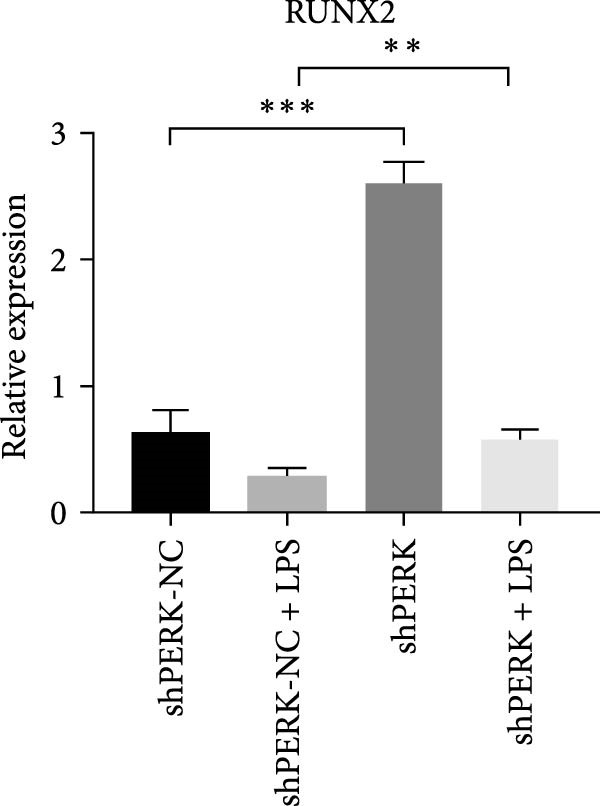
(J)

**Figure:**
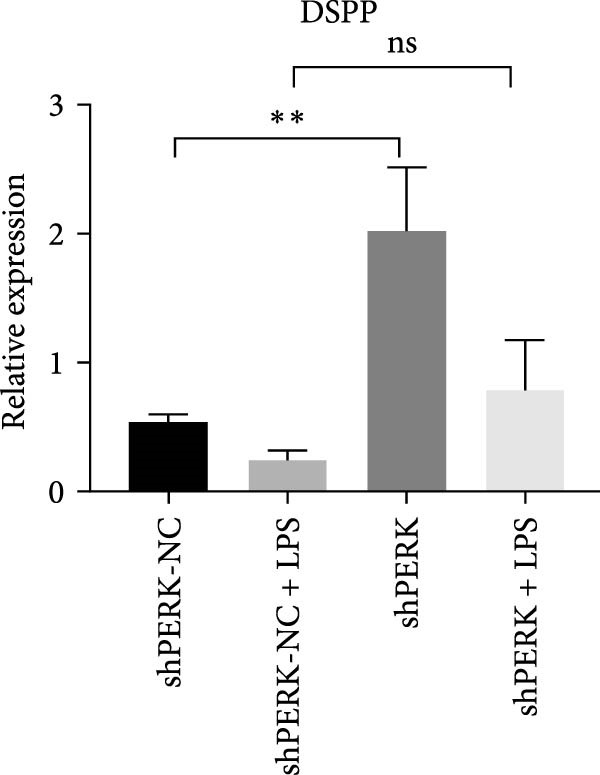
(K)

**Figure:**
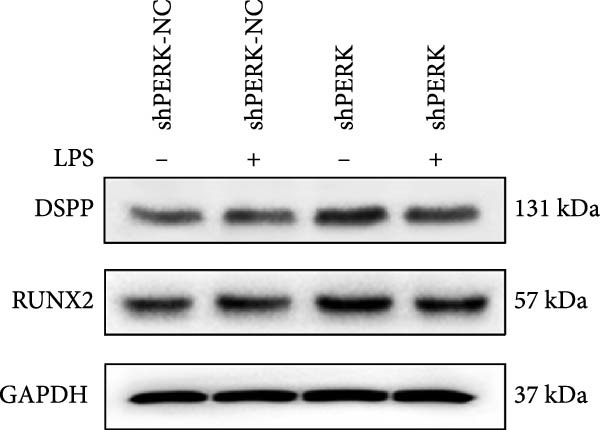
(L)

**Figure:**
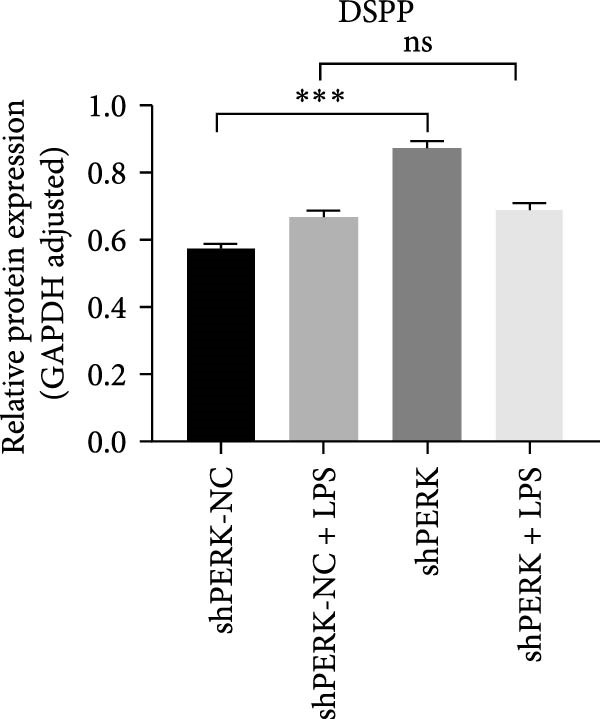
(M)

**Figure:**
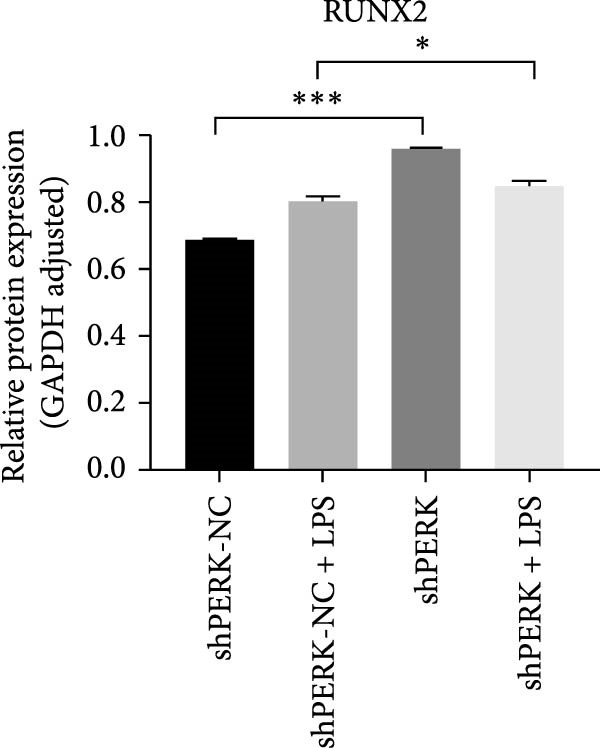
(N)

**Figure:**
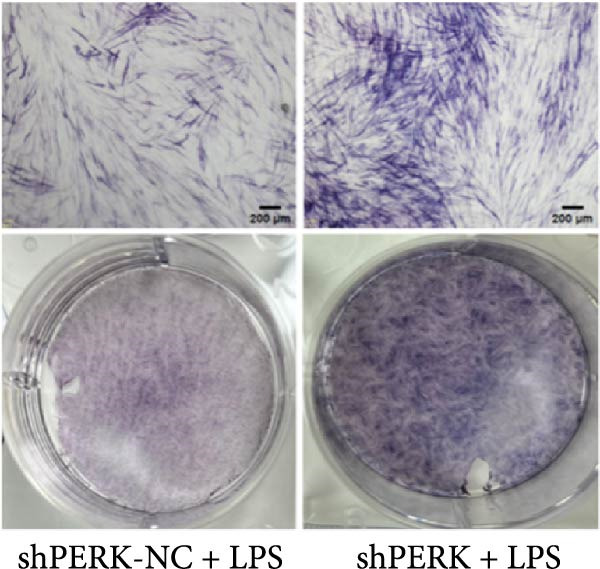
(O)

**Figure:**
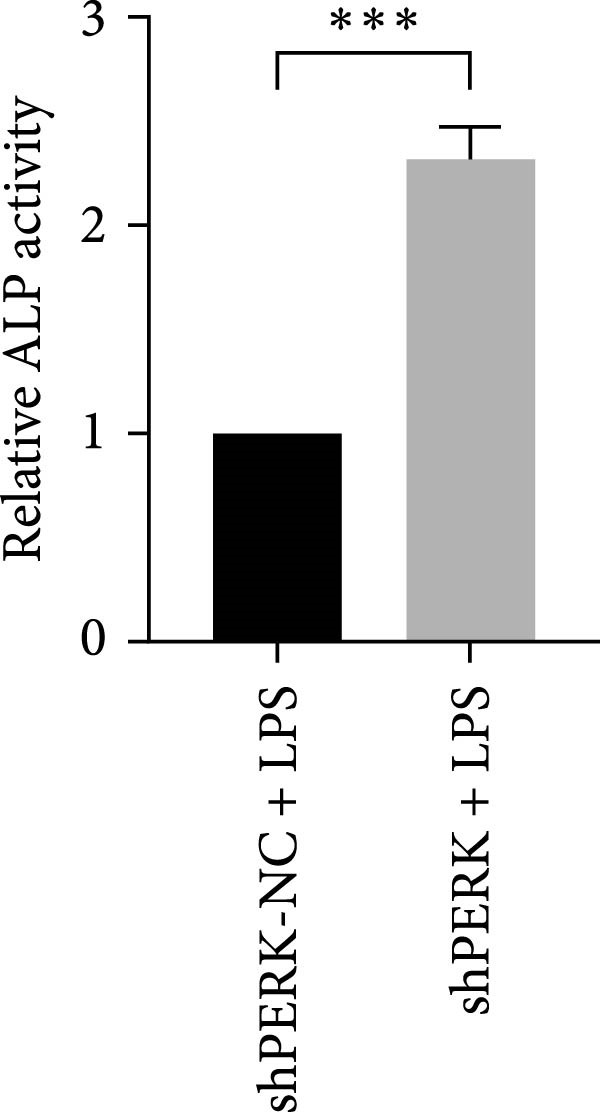
(P)

**Figure:**
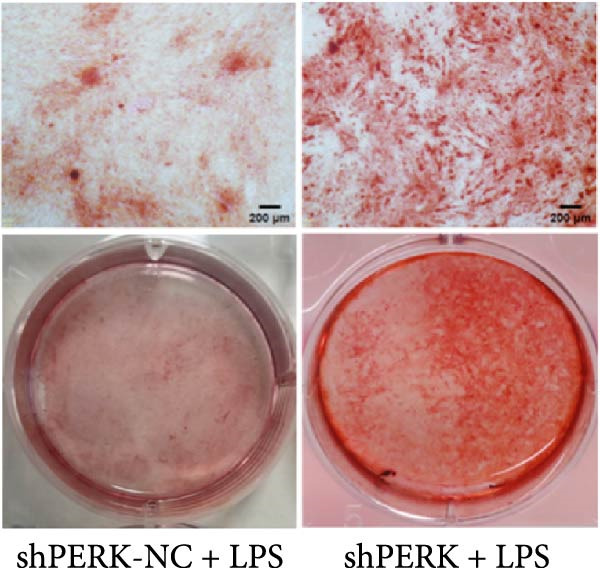
(Q)

**Figure:**
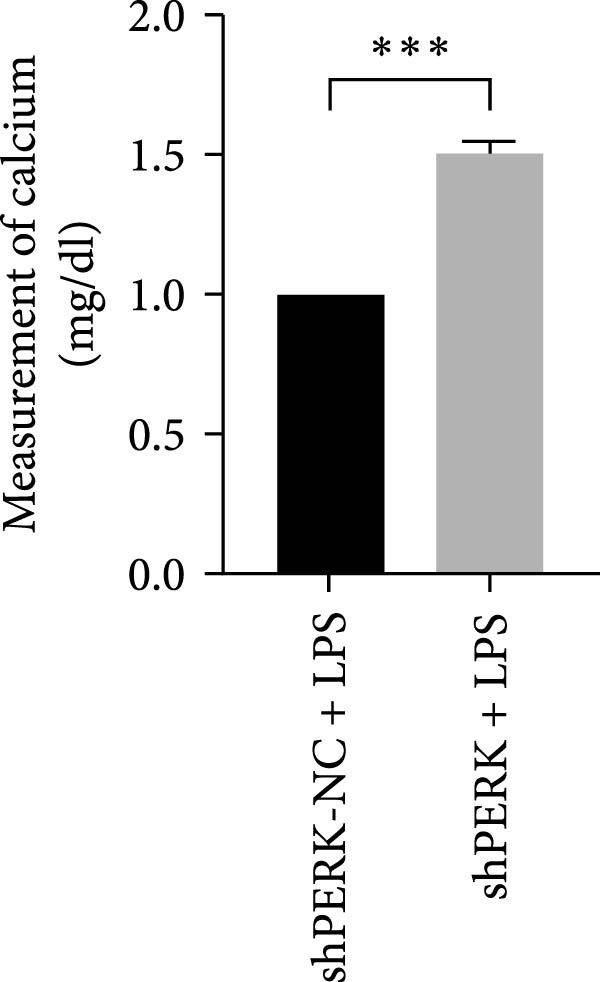
(R)

### 3.4. PERK Might Regulate Odontogenesis Through the Mitochondrial‐Associated Endoplasmic Reticulum Membrane (MAM)

To explore the underlying mechanisms of the regulatory effects of PERK on odontogenesis, RNA‐Seq was performed following treatment of hDPSCs with 20 μg/mL LPS for 6 h. Among the identified genes, 4176 upregulated and 1827 downregulated genes were identified in the shPERK group compared to the control group (Figure 4A). The top enriched GO terms include protein modification (phosphorylation and ubiquitination), protein transport, lipid metabolism, plasma membrane, endoplasmic reticulum (ER), mitochondria, protein binding, and metal ion binding on the 6003 differentially expressed genes (Figure 4B). Meanwhile, KEGG demonstrated pathways in cancer, ribosome, PI3K‐Akt signaling, and Calcium signaling; MAPK signaling pathway may also be involved (Figure 4C). Moreover, PERK knockdown may influence the physiological functions of MAM, including lipid metabolism and Ca^2+^ homeostasis [10].

Figure 4Regulatory mechanism of PERK analyzed using RNA‐Seq. (A) Differentially expressed genes between the shPERK and control groups. (B) GO enrichment analysis of the differentially expressed genes. (C) KEGG pathway enrichment analysis of the differentially expressed genes.

**Figure:**
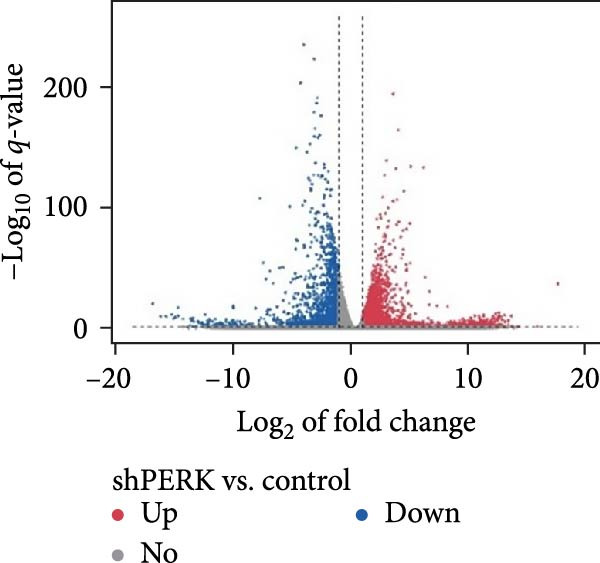
(A)

**Figure:**
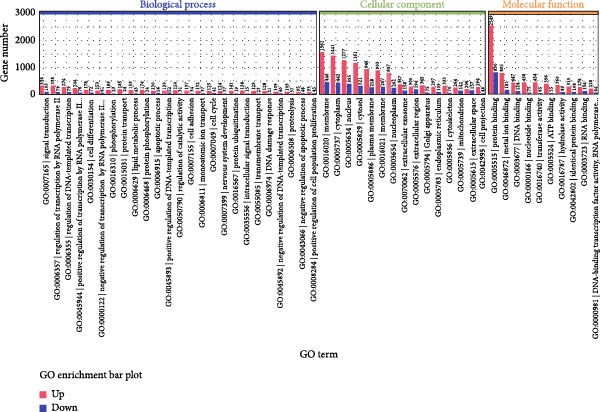
(B)

**Figure:**
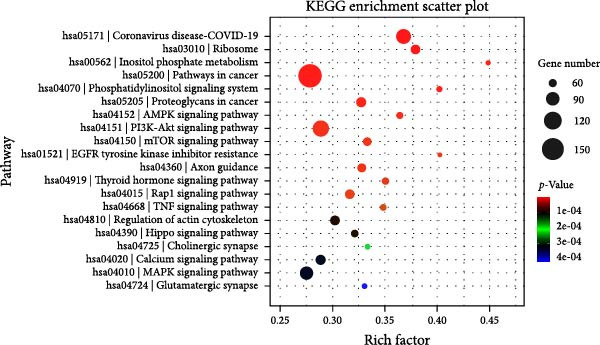
(C)

## 4. Discussion

Maintaining hDPSCs homeostasis and improving their biological activity can aid in the self‐repair and regeneration of injured PDC [9, 11]. Studies have suggested the involvement of the ERS–PERK pathway in the mineralization process [5, 12, 13], such as osteoblasts derived from Perk^−/−^ mice, which exhibited diminished mineralization in cell cultures and defective collagen‐processing capabilities [14].

In this study, we confirmed the involvement of PERK in PDC regeneration, especially during the initial in vivo stage. However, the specific relationship between ERS–PERK and odontoblastic differentiation of hDPSCs during PDC injury and regeneration requires further investigation.

As a principal cellular organelle, the ER is integral to a multitude of metabolic processes, such as protein secretion, transmembrane protein folding, calcium homeostasis, and lipid metabolism, and plays a critical role in sensing cellular stress and maintaining homeostasis. Various disturbances, such as oxidative stress, genetic mutations, and free fatty acids, have been shown to perturb cell homeostasis, leading to ERS [3]. A mutual coupling effect exists between the ERS and the inflammatory response; ERS participates in physiological adaptive responses during inflammation to maintain cellular homeostasis [15, 16]. Our results showed that moderate concentrations of LPS stimulus (10 and 20 μg/mL for 6 h) could induce PDC injury and markedly upregulate PERK and ATF4, confirming that inflammation could activate the ERS–PERK pathway. Several studies have suggested that proinflammatory cytokines induce ERS via ROS accumulation and Ca^2+^ release, which cause the accumulation of unfolded or misfolded proteins within the ER lumen and mitochondrial dysfunction [17, 18]. However, the precise mechanisms underlying this process remain to be elucidated.

Our in vivo findings demonstrated a distinct temporal and spatial activation pattern of PERK during pulpal injury. PERK can potentially facilitate the reparative response, but significantly decreases at later stages, accompanied by severe inflammation and tissue necrosis. This differential expression suggests PERK as a stress‐responsive mediator that maintains cellular homeostasis and promotes repair processes under mild injury conditions. In contrast, its dysfunction or downregulation under severe inflammation may be associated with impaired regenerative capacity. Further investigations remain warranted to elucidate the precise role or balance of PERK in coordinating the adaptive and apoptotic responses during PDC injury and repair.

The ERS and inflammatory responses constitute a dual‐edged sword in intricate biological processes [19]. Clinically, mild stimuli can serve as an adaptive response to remove noxious stimuli and lead to a repair reaction to protect against inflammatory damage [20]. In contrast, intense insults affect the fate of cells and the outcome of cell‐mediated tissue reparative or regenerative endeavors [21]. Similarly, our results showed that excessive LPS (10 and 20 μg/mL) could disrupt odontogenic differentiation of hDPSCs, trigger PERK activation, and induce apoptosis of hDPSCs. After knocking down PERK, the levels of apoptosis‐related genes (CHOP, JNK, caspase‐12) induced by excessive LPS (10 μg/mL, 6 h) decreased, while the odontogenic ability of hDPSCs was rescued. Studies reported the phenomenon may attribute to interruption of several canonical pathway such as Notch, BMP/Smad and Wnt/*β*‐catenin signaling [22–24]. Moreover, the ERS–PERK pathway may converge into a proapoptotic network in which CHOP upregulates proapoptotic BH3‐only proteins while suppressing the antiapoptotic lymphoma‐2 (BCL2) protein.

hDPSCs are a promising stem‐cell type for regenerative therapies that require a series of accurate protein quality control mechanisms to synthesize and secrete proteins [25]. ERS is closely related to osteogenesis imperfecta (OI) and rheumatoid arthritis (RA), suggesting that ERS is a cellular response to defective collagen trafficking and osteoblast dysfunction [26]. The ERS–PERK pathway is involved in odontogenic/osteoblastic differentiation [14]; however, the regulation of ERS–PERK activation in odontogenic/osteoblastic differentiation of MSCs remains controversial. Some studies have affirmed that PERK overexpression enhances the osteogenic capacity of human PDLCs (hPDLCs), characterized by elevated ALP activity and osteogenic genes [27], while its depletion produces the opposite changes [7]. Conversely, another study reported that stem cell differentiation in various tissues was mediated by PERK‐dependent mRNA translation suspension, and the induction of ERS caused a stemness loss of intestinal stem cells in a PERK‐eIF2*α*‐dependent manner [28]. In alcohol‐induced ERS, Wnt/*β*–catenin pathway suppression and activating TNF‐*α* signaling via PERK/ATF4/CHOP pathway inhibit osteogenesis of MSCs [29]. Our study also revealed that ERS–PERK was activated by LPS stimulus (10 and 20 μg/mL, for 6 h), and PERK knockdown could upregulate odontoblastic differentiation markers in hDPSCs under LPS stimulus (10 μg/mL). This indicates that the PERK pathway may suppress odontogenic ability under physiological or inflammatory conditions. Therefore, PERK exhibits dual properties and can potentially advance therapeutic interventions, considering how the ERS–PERK pathway can be precisely regulated to create a supportive microenvironment for odontogenesis.

Several key considerations regarding the inhibitory effect of PERK on odontogenesis should be taken into account. Initially, inflammatory cytokines disrupt UPR function through MORF‐mediated PERK transcription, which contributes to impaired ER function, prolonged ERS, and defective osteogenic differentiation of PDLSCs in periodontitis‐associated chronic inflammation [30]. Second, a baseline level of CHOP is necessary for osteoblast differentiation [26]. Herein, the apoptosis rate of hDPSCs was consistently higher than the survival rate with LPS stimulation for 72 h at 10, 20, 30, 40, and 50 μg/mL. PERK knockdown downregulated apoptosis‐related genes (CHOP, JNK, and caspase‐12) and upregulated odontogenic differentiation of hDPSCs. It is plausible that the decrease in CHOP expression induced by PERK knockdown reached a certain extent beneficial for odontogenic differentiation.

Thus, inhibiting the ERS–PERK pathway during inflammation may have significant potential for PDC regeneration.

RNA‐Seq was used in this study to further explore the potential molecular mechanisms contributing to the observed enhancement of osteogenesis following PERK knockdown. The involvement of MAM in this biological process has been proposed. MAM, a multifunctional microdomain in cellular homeostasis, is involved in various cellular processes, including calcium homeostasis, mitochondrial dynamics, and lipid metabolism [31]. Structurally, PERK operates at some capacity at the MAM. At the molecular level, PERK transfers ROS signals from the ER to the mitochondria and interacts with two proteins (vesicle‐associated membrane‐associated proteins [VAPA/B] and sarcoplasmic/ER Ca ATPase 2 [SERCA2]) involved in Ca^2+^ handling and ER‐mitochondria contact sites, independent of its canonical role [32]. Further research is warranted to determine whether MAM is a bridge between PERK and hDPSCs’ odontogenesis and elucidate the underlying mechanisms.

The ERS–PERK pathway exerts a notable effect on inflammatory processes and odontogenesis in hDPSCs. Future research should focus on elucidating the specific mechanism, especially in terms of detailed signaling cascades, and the role of the ERS–PERK pathway in modulating the immune response of hDPSCs under inflammatory conditions. Clarifying this intricate regulatory network will deepen our understanding of the mechanisms underlying odontogenesis and inflammatory responses involving both pro‐ and anti‐inflammatory effects. Moreover, the development of local drug delivery systems loaded with small molecules targeting the ERS–PERK pathway may enhance the odontogenic differentiation and regenerative therapeutic effects of hDPSCs in the inflammatory microenvironment.

## 5. Conclusion

This study shows that LPS activates the ERS–PERK pathway, increases the expression of inflammatory factors, and inhibits the odontogenic ability of hDPSCs. PERK knockdown can accelerate the deposition of Ca^2+^ and the matrix mineralization process, thereby promoting the osteogenic differentiation of hDPSCs in the inflammatory microenvironment simulated by LPS.

## Disclosure

All the authors have read and approved the final version of the manuscript.

## Conflicts of Interest

The authors declare no conflicts of interest.

## Author Contributions

Ruirui Liu, Lin Niu, and Huimin Li conceived the study, participated in its design and coordination, and revised the manuscript critically. Yun Yang and Huimin Li searched the databases. Yun Yang, Huimin Li, Yating Miao, and Zhipu Luo completed all the experiments. Yun Yang and Yating Miao analyzed the data and drafted the manuscript. Ruirui Liu and Lin Niu reviewed and edited the manuscript. Yun Yang and Huimin Li are co‐first authors.

## Funding

This work was supported by the Natural Science Foundation of Shaanxi Province (Grant 2022JZ‐56).

## Supporting Information

Additional supporting information can be found online in the Supporting Information section.

## Supporting information


**Supporting Information** Figure S1. (A) Spindle shaped hDPSCs crawled out of the tissue block at 7–10 days and arranged in a vortex shape with uneven sizes and visible impurities. (B) After passaged, the morphology and size of hDPSCs were more uniform and consistent, and the arrangement was also more compact. (C) Flow cytometric analysis indicated hDPSCs strongly express CD90 and CD29, but negative for CD45. Figure S2. (A, B) ALP staining and quantitative analysis of hDPSCs cultured in NC and OM stimulated by different concentration of LPS. (C, D) Alizarin red S staining and quantitative analysis of hDPSCs cultured in NC and OM stimulated by different concentration of LPS. (significance was determined via Student’s *t* test analysis of variance; data are represented as mean ± SD;  ^∗^ *p* < 0.05,  ^∗∗^ *p* < 0.01,  ^∗∗∗^ *p* < 0.001) Figure S3. hDPSCs were transfected by lentivirus at multiplicity of infection (MOI) at 1, 10, 20, 50, and 100 with or without polybrene. Green fluorescence could be detected 72 h after transfection.

## Data Availability

The data that support the findings of this study are available from the corresponding author upon reasonable request.
