# Supplementary material for: ERS–PERK Pathway is Involved in the Repair of the Pulpo‐Dentinal Complex Under an Inflammatory Microenvironment
Source: Stem Cells Int. 2025 Dec 11;2025:7689998. doi: 10.1155/sci/7689998 (PMC12767467; doi:10.1155/sci/7689998)
Supplement: Supplementary file 1 — Supporting Information Figure S1. (A) Spindle shaped hDPSCs crawled out of the tissue block at 7–10 days and arranged in a vortex shape with uneven sizes and visible impurities. (B) After passaged, the morphology and size of hDPSCs were more uniform and consistent, and the arrangement was also more compact. (C) Flow cytometric analysis indicated hDPSCs strongly express CD90 and CD29, but negative for CD45. Figure S2. (A, B) ALP staining and quantitative analysis of hDPSCs cultured in NC and OM stimulated by different concentration of LPS. (C, D) Alizarin red S staining and quantitative analysis of hDPSCs cultured in NC and OM stimulated by different concentration of LPS. (significance was determined via Student’s t test analysis of variance; data are represented as mean ± SD; ∗ p < 0.05, ∗∗ p < 0.01, ∗∗∗ p < 0.001) Figure S3. hDPSCs were transfected by lentivirus at multiplicity of infection (MOI) at 1, 10, 20, 50, and 100 with or without polybrene. Green fluorescence could be detected 72 h after transfection. [file SCI-2025-7689998-s001.docx]

**Supplement:**


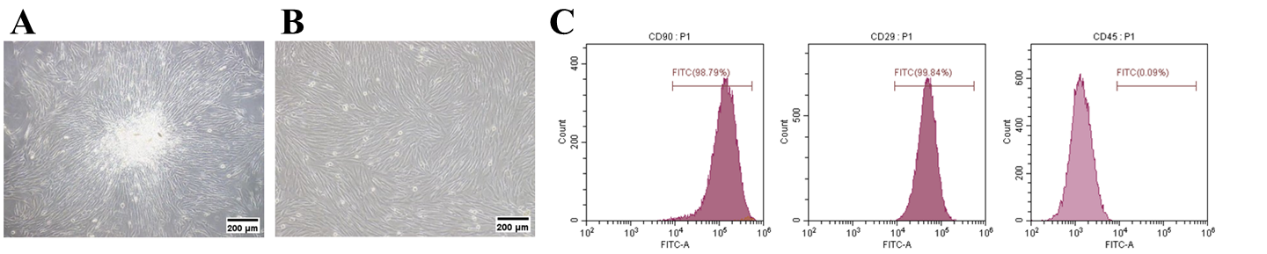


Fig. S1. (A) Spindle shaped hDPSCs crawled out of the tissue block at 7-10 days and arranged in a vortex shape with uneven sizes and visible impurities. (B) After passaged, the morphology and size of hDPSCs were more uniform and consistent, and the arrangement was also more compact. (C) Flow cytometric analysis indicated hDPSCs strongly express CD90 and CD29, but negative for CD45.


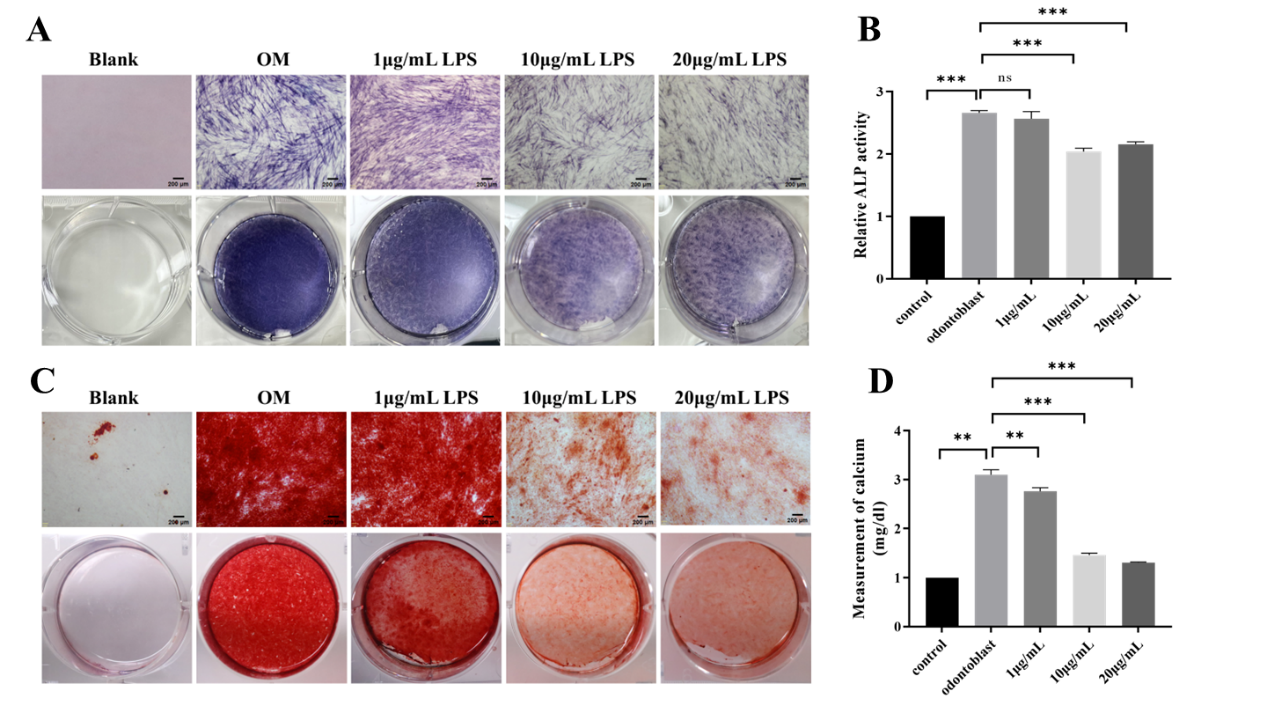


Fig. S2. (A-B) ALP staining and quantitative analysis of hDPSCs cultured in NC and OM stimulated by different concentration of LPS. (C-D) Alizarin red S staining and quantitative analysis of hDPSCs cultured in NC and OM stimulated by different concentration of LPS.

(Significance was determined via Student’s *t*test analysis of variance; data are represented as mean ± SD. **P* < 0.05. ***P* < 0.01. ****P* < 0.001.)


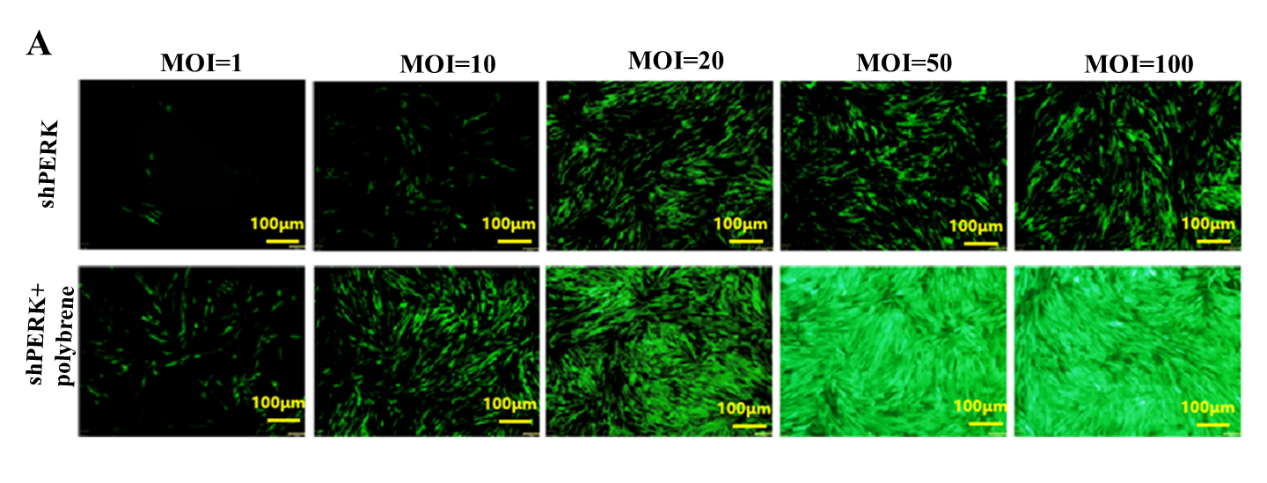


Fig. S3. hDPSCs were transfected by lentivirus at multiplicity of infection (MOI) at 1, 10, 20, 50, 100 with or without polybrene. Green fluorescence could be detected 72 h after transfection.
